# Historical Perspectives on the Development of Current Standards of Care for Enzymatic Debridement

**DOI:** 10.3390/medicina56120706

**Published:** 2020-12-17

**Authors:** Wolfram Heitzmann, Paul Christian Fuchs, Jennifer Lynn Schiefer

**Affiliations:** Clinic of Plastic, Reconstructive, Hand and Burn Surgery, Hospital Cologne Merheim, University of Witten-Herdecke, 51109 Cologne, Germany; fuchsp@kliniken-koeln.de

**Keywords:** burn therapy, enzymatic debridement, bromelain, NexoBrid^™^

## Abstract

*Background and Objective:* The use of plant-based products for burn treatment dates back to 1600 BC. Enzymatic debridement, which can be achieved as non-surgical or conservative debridement, has recently gained increasing attention. Several reviews have been published thus far. However, there has been no historical article including the achievements of the last 20 years, and this is the first review to present the achievements made in the field of enzymatic debridement in the last 20 years. This study aimed to present a historical overview of the development of enzymatic debridement until the present day. *Methods*: Enzymes from bacteria and plants were initially used for full-thickness burn treatment; however, they did not gain attention. Papain-derived products were the first plant-based products used for enzymatic debridement. Sutilains gained broad use in the 70s and 80s but came off market in the 1990s. Bromelain has been used for burn treatment owing to its strong debriding properties. NexoBrid^™^ is used as a minimally invasive approach for enzymatic debridement of deep dermal burns. However, its use has been limited due to commercially available bromelain and the presence of four distinct cysteine proteinases. NexoBrid^™^ involves faster eschar removal together with reduced blood loss, leading to improved long-term outcomes. However, research on nonoperative enzymatic debridement of burns has taken decades and is still ongoing. *Results*: Overall, the results of our study indicate that necrectomy, which has been used for a long time, remains the standard of care for burns. However, enzymatic debridement has several advantages, such as faster eschar removal, reduced blood loss, and reduced need for skin grafting, especially in cases of facial and hand burns. Enzymatic debridement cannot replace surgical intervention, as the enzyme only works on the surface of the eschar. Enzymatic debridement is not recommended in the early phase of scald burns. *Conclusions*: Enzymatic debridement has become an integral part of burn therapy and the standard of care in specific burn centers.

## 1. Introduction

Burn injuries have always been and remain frequent, and burn therapy is a highly challenging field of medicine. In 2004, nearly 11 million burn injuries reported worldwide were severe, requiring medical attention [1]. Fortunately, a vast majority of burns are not fatal because of high standards of medical care and progress in modern burn therapy. The first and most important step in burn therapy is the total removal of eschar to avoid critical complications such as wound infections or compartment syndromes and to initiate wound healing. The first reference to debridement dates back to 25 AD, when the Roman encyclopedist Aulus Cornelius Celsus described a surgical wound treatment with operative removal of the burned skin [2]. Since 1970, early excision of burns has been considered the standard therapy [3].

However, this debridement method had disadvantages such as causing huge trauma and excessive bleeding, as well as having insufficient and selective removal from the burned region [4]. Therefore, a search for alternative debridement methods was initiated, and techniques such as laser-induced thermotherapy and the use of water jet surgical tools (e.g., Versajet) entered the domain of eschar removal. With these new debridement techniques, the vital dermal tissue and, more importantly, stem cells could be preserved to a greater extent. This, in turn, could lead to higher rates of spontaneous re-epithelialization and improved healing and scar quality. The most selective form of debridement can be achieved by non-surgical or conservative debridement. Among the techniques used for this method, enzymatic debridement has gained the most attention in recent decades. The first products used for enzymatic debridement were plant-based ones, starting with Papain-derived products in 1940. Others like Ficin or Debricin, enzymes made of ficus carica, and Bromelain, made of ananas comosus, followed. On the other hand, efforts were made to use products of bacterial origin such as Streptokinase from Hemolytic streptococci, Santyl from *Clostridium histolyticum,* or Travase from Bacillus subtilis. 

The use of bromelain-based enzymatic debridement (NexoBrid^™^, Mediwound, Isreal) has become an integral part of burn therapy and the standard of care (SOC) in specific burn centers, especially in cases of facial and hand burns [5]. In light of the above, it is worth looking back on the history of enzymatic debridement to understand its role in modern medicine and simultaneously glance forward to the promising prospects that lie ahead in the field of burn therapy. Thus far, several reviews have been published. However, this study is the first to include the achievements made in the field of enzymatic debridement in the last 20 years. This study aims to present a historical overview of the developments in enzymatic debridement until the present day.

## 2. Different Sources of Enzymes

The use of plant-based products for burn treatment dates back to 1600 BC. The Egyptian Smith Papyrus describes the use of resin and honey for treating burn wounds. By 1500 BC, other herbal remedies such as *Cyperus esculentus* had been added to the list of substances for treating burns [6]. However, it was not until 1940 that enzymes of plant origin were used for eschar removal. At first, papain was extracted from the juice made using the fruits and leaves of *Carica papaya*. Papain was activated by adding either triethanolamine [7] or cysteine hydrochloride with sodium salicylate [8]. All of these solutions had a strong debriding effect. Guzman et al. used papain solution on wet surgical gauze for dressing burn wounds without any additional activator and achieved satisfactory debridement results [9]. In addition, an enzyme made from fig tree latex (debricin) showed a rapid debridement effect on second-degree burns; however, no further investigation was performed due to lack of standardization [10]. Currently, bromelain-based products are commonly used in most parts of the world. (Appendix A).

Another group of enzymes with debriding properties has bacterial origin. In 1951, Altemeier et al. described enzymes derived from *Clostridium histolyticum*, *Escherichia coli*, *Pseudomonas aeruginosa*, and *Bacillus proteus*. In vitro and in vivo collagenase made from *Clostridium histolyticum* showed the most potent effects [11]. The only such product with Food and Drug Administration approval in the USA is clostridial collagenase ointment (CCO) (Santyl). There is evidence for CCOs’ positive effect on burn wounds [12]. The findings suggest that CCO can be used to debride burn wounds with less pain and nursing labor than traditional therapy with other silver-impregnated products. However, large randomized controlled trials are needed in the future to draw definitive conclusions. In contrast, streptokinase and streptodornase (Varidase) showed disappointing results, especially in the case of debridement of full-thickness burns. This is why they have not gained acceptance in burn therapy [13].

Garret was the first to publish a study on neutral proteases made from *Bacillus subtilis* (sutilains) in 1969. Over 100 patients were treated efficiently using sutilains [14]. Under the tradename Travase, sutilains gained increasing attention in the 1970s and 1980s. However, treatment with Travase led to an increasing number of wound infections soon after the application of the enzyme. A possible postulated reason for this side effect was the need for a moist environment, which stimulates bacterial growth [15,16]. To compensate for this adverse effect, simultaneous treatment with antiseptic substances such as silver sulfadiazine or mafenide is recommended. Moreover, depending on the debriding effect in that case, the patient needs to be treated in a moist environment for 3–10 days, which is a long treatment time. The debridement effect can be accelerated by applying Travase twice a day instead of once and by starting application on day 1 postburn. Using this process, full debridement can be obtained within 24 h. Wound closure can be achieved faster by autologous skin grafting than with standard conservative treatment. This made Travase the most commonly used enzyme in American burn units until it came off market in the 1990s [17,18].

Another non-surgical method for the debridement of eschar involved the use of acids, mainly pyrovic acid and phosphoric acid, until the 1960s [19,20]. An obvious disadvantage of this therapy was uncomfortable, painful, and long-lasting debridement. Therefore, this approach was abandoned and replaced by early surgical eschar removal in the 1970s.

During 1965–1979, several scientific groups examined additional enzymes such as trypsins, chymotrypsins, and fibroylsin-desoxyribonuclease. These enzymes prevented wound infection but did not reach relevant clinical use [21]. Vibriolysin extracted from *Vibrio histolyticus* and blowfly larvae extracts met the same fate [22,23].

Searching for an agent that could supersede surgical debridement, Klasen et al. reported in 2000 that chemical or enzymatic debridement had not yet achieved the status of general application. The main reasons were poor quality, high variability of composition, and lack of standardization of enzymatic treatment [24]. With the help of novel technologies in enzyme extraction and processing, these obstacles have now been overcome.

In the field of burn research, bromelain has gained the most attention during the last decade. Thus, it is the only enzyme that has achieved general application in Europe. Therefore, it is worth taking a closer look at its past, present, and future.

## 3. The Discovery of Bromelain

The preparation of a new protease mixture from the pineapple plant was described for the first time by Heinicke and Gortner in 1957. Batting of hides, tenderizing of meat, or chill-proofing of beer were the initially considered non-medical areas of application. Furthermore, these authors coined the term “bromelain”, indicating any protease made from any member of the Bromeliaceae family [25]. 

The rise of the use of bromelain for burn treatment was noted in 1971, when Levine et al. tested the debriding effect of several different enzyme mixtures in vitro. They found that bromelain had the strongest debriding properties when assessed on the basis of hydroxyproline release. Further, they observed that adding mafenide did not inactivate bromelain’s debriding effect, whereas sulfadiazine did [26]. They continued their experiments on bromelain in 1973 on a porcine model with third-degree burns. Again, bromelain showed a good debriding effect without converting second-degree burns into third-degree burns and without having any local toxic effects on the pigs [27]. 

Levenson et al. also studied bromelain using different animal models [28]. However, the bromelain available at the time consisted of a mixture of proteolytic enzymes with an unknown chemical composition. Thus, there was no reproducibility or standardization [29]. Different substances were then added to reinforce the debriding effect of bromelain. Therefore, bromelain was combined with several mercaptans, such as N-acetyl cysteine, penicillamine, and cysteine ethyl ester. Application of N-acetyl cysteine to deep burns resulted in faster healing than applying conventional treatment to burns in rats. They concluded that mercaptans have debriding properties, act quickly, are not toxic, and reinforce the debriding effect of bromelain when combined with it [24,30,31]. However, the use of this combination has not prevailed.

Klein et al. published the first relevant clinical trial investigating the treatment of burn patients with bromelain in 1985. They described varying debridement success, most likely due to the differences in the composition of the preparations because of botanical variations [24,32]. At the same time, Boswick et al. investigated enzymatic debridement with bromelain in a multi-center study in the USA. At three burn centers, 36 patients were debrided with bromelain. Adequate debridement took up to 24 h in nearly half of the patients. The rest required additional surgery because of insufficient enzymatic debridement. The possible reasons for debridement failure were delayed application of the enzymes and pretreatment with silver sulfadiazine [33].

## 4. Pitfalls during the Implementation of Enzymatic Debridement with Bromelain

After being tested in several in vitro models and animal models, bromelain finally reached clinical trials in 1985. However, these first clinical findings were less promising than expected, showing insufficient debriding effects. One cause worth mentioning was the inactivation of bromelain by the commonly used silver sulfadiazine [26]. However, the most important reason for inconsistent findings in these studies was the use of commercially available bromelain that was not standardized, and there was no measurable enzyme composition. The presence of at least four distinct cysteine proteinases, namely, ananain1, ananain2, stem bromelain, and comosain, has been described [34]. Therefore, each patient within a trial was treated with an agent with different proteolytic enzyme composition with different debriding properties, leading to variations in debriding intensity. With the commercial production of NexoBrid^™^, made from pineapple stems, a product with a standardized bromelain composition was finally available.

## 5. Enzymatic Debridement with NexoBrid^™^

### 5.1. First Advantages of Nexobrid over SOC

Since 18 December 2012, NexoBrid^™^ has gained approval as a minimally invasive technique for enzymatic debridement of deep dermal burns in Europe. The first multicenter study on the product was published in 2014 by Rosenberg et al. They showed that enzymatic debridement with NexoBrid^™^ resulted in faster eschar removal with reduced blood loss than the SOC. Furthermore, a reduction in the need for autografting was achieved because of more selective debridement, which spared the vital dermis. This again led to a reduction in donor site morbidities while achieving comparable long-term results of wound healing in esthetics, function, and quality of life [35]. 

### 5.2. The Learning Curve

In 2017, Schulz et al. demonstrated their initial learning curve in the enzymatic debridement of severely burned hands using NexoBrid^™^. Twenty patients with deeply burned hands were treated with NexoBrid^™^. The treatment was efficient in 90% of the cases. Correct wound-bed evaluation was described to be challenging, and wound-bed appearance was found to be different from surgical excision. Therefore, surprisingly, the majority of the burn surface areas were overestimated. Treatment was performed under plexus anesthesia by one burn surgeon and one nurse. With this new process, the treatment costs could be significantly reduced. Although these patients had sustained deep burns on their hands, there was no need for skin grafting after enzymatic debridement. Suprathel was used as a wound dressing. In this study, the mean number of days required for complete wound healing was 28 [36]. 

### 5.3. Less Need of Autografting at Same Scar Quality Compared to SOC

Thus far, excisional debridement with autografting has remained the SOC for burn therapy. Because of the promising findings on enzymatic debridement for burned hands, Schulz et al. compared the SOC to enzymatic debridement with bromelain (NexoBrid^™^, EDNX). Therefore, 20 patients with deep-dermal or full-thickness burns on the hands were treated with surgical excision of the necrotic tissue, whereas 20 patients with similar burns were treated with NexoBrid^™^. EDNX was superior in burn-depth evaluation, tissue preservation, completeness of debridement, and wound closure. The number of wounds requiring autograft was reduced for those treated with NexoBrid^™^. However, scar quality after 3 months did not differ substantially between the two groups [37].

### 5.4. European Guidelines 

To summarize, there is increasing evidence that enzymatic debridement is a powerful method for eschar removal in burn wounds, reducing blood loss, need for autologous skin grafting, and need for surgical excision. To assess the role and clinical advantages of NexoBrid^™^ beyond the scope of the literature and in view of the users’ experience, a European consensus meeting was scheduled. The first European guidelines on the use of NexoBrid^™^ were set in 2017 by Hirche et al. based on their experience of applying this enzymatic debridement in more than 500 patients [38].

In 2017, Loo et al. evaluated the evidence in published studies on the benefits of using NexoBrid^™^ compared with the use of traditional surgical excision (the SOC) for burn wound debridement. Studies published from 1986 to 2017 were considered. They confirmed strong supporting evidence of the superior effect of NexoBrid^™^, based on the time needed for complete debridement, need for surgery, area of burn excised, and need for autografting. Anecdotal and refuting evidence was found only for the proposed improvement in scar quality and reduced time needed for wound healing [39].

With growing experience in the use of enzymatic debridement, especially for burns of the hand, face, and the genital area, Hirche et al. released a consensus guideline update in 2020 based on the clinical experience of and practice patterns followed for 1232 summarized cases. The degree of consensus (97.7%) was remarkably high. This alone shows the success and significance of enzymatic debridement therapy in burn treatment. However, Hirche et al. reported that surgical excision with tangential knives and/or hydro-surgery remains the SOC [5]. In addition to all these positive effects, it must be outlined that in case of long-term results, such as esthetics, function, and quality of life, the effects of SOC and enzymatic debridement are comparable.

### 5.5. Limitations of the Use of Nexobrid

With increasing experience in enzymatic debridement, some limitations have also been uncovered.

There have been implications that enzymatic debridement does not work well on burned feet within cases of established diabetic foot disease. In a study on such cases, all patients experienced wound deepening post enzymatic debridement and needed additional surgical necrectomy, most likely due to microangiopathy [40]. To date, there is no concrete knowledge about the effects of enzymatic debridement in chemical burns, which requires further research. Furthermore, there is a consensus that enzymatic debridement should not be used as therapy for high-voltage injuries. In patients with this injury pattern, deep muscle damage with increasing compartment pressure is likely if enzymatic debridement is performed. Enzymatic debridement cannot replace surgical intervention with compartment release in these cases, as the enzyme works only on the surface of the eschar. Additionally, enzymatic debridement is not recommended in the early phase of scald burns, as poor results have been shown in these cases. Finally, there have been only a few instances of enzymatic debridement performed on large surfaces. Thus far, this attempt seems to be realizable, but the systemic effects of bromelain on patients with large-surface burns and the effects of enzymatic debridement on water loss and volume management should first be evaluated [5]. 

## 6. Conclusions

Since the Second World War, investigation into nonoperative enzymatic debridement of burns has been ongoing. Several enzymes and other chemical agents have been tested worldwide for their debriding properties [24]. However, results have been highly variable because enzyme compositions have been neither constant nor reproducible. Additionally, enzyme quality has been low, as the production methods have not been technologically advanced enough. Therefore, it was not until 2013 that with NexoBrid^™^, the first agent with a well-known and constant composition of enzyme preparation, that enzymatic debridement of burns achieved general application within Europe [35].

The most important advantage of enzymatic debridement is that the selectivity of enzymes toward damaged and unsalvageable tissue is greater than that of mechanical eschar removal. By saving the vital dermis and stem cells, higher rates of spontaneous re-epithelialization have been achieved and the need for autografting has been reduced. This leads to reduced donor site morbidity. Particularly in areas with thin subcutaneous tissue, where relevant structures are left vulnerable, the advantages of NexoBrid^™^ are essential. To achieve the best results, enzymatic debridement is followed by the use of resorbable skin substitutes, such as Suprathel. However, further investigation in the field of wound treatment after enzymatic debridement is awaited. Another advantage of enzymatic debridement of burns is the prevention of operative escharotomy in circumferential deep burns of the distal upper extremity [41]. For this, the NexoBrid^™^ treatment must be initialized immediately, omitting the presoaking phase [5].

**Limitations:** It must be mentioned that this study represents a review of literature. The articles included in the study were selected according to the PRISMA flow diagram. Several records found in the database search were excluded due to lack of relevance in the historical overall context or lack of availability. This could be considered as a limitation of this study.

Taking the whole history of enzymatic debridement into consideration, we can draw conclusions that research on its application has already gained increasing attention. Enzymatic debridement has become an integral part of burn therapy and the SOC in specific burn centers. However, further investigations into some of the areas mentioned above is needed to allow enzymatic debridement to reach its full potential.

## Appendix A

**Table A1:** Overview of debriding enzymes [7,10,11,13,14,25].

	Most Important Enzymes	Publication Date	Author	Enzyme Source	Advantages	Disadvantages
Plant origin	Papain	1940	Glasser	*Carica papaya*	Strong debriding effect	Activator substance needed (e.g., triethanolamine)
Plant origin	Ficin/Debricin	1949	Connel	*Ficus carica*	Rapid debriding effect	Lack of standardization
Plant origin	Bromelain	1957;1985	Heinicke, Klein	*Ananas comosus*	Fastest debriding effect, high level of standardization approval as a medical product (NexoBrid™)	Expensive treatment standard use only in burn centers due to the learning curve
Bacterial origin	Clostridial collagenase ointment/CCO/Santyl	1951	Altemeier	*Clostridium histolyticum*	Effective debridement of human eschar	Lack of randomized trials
Bacterial origin	Streptokinase/Streptodornase/Varidase	1952;1957	Teitelman, Connel	Hemolytic streptococci	-	Insufficient debridement of full-thickness burns
Bacterial origin	Sutilains/Travase	1969	Garett	*Bacillus subtilis*	Wound closure by autologous skin grafting is achieved faster than that with SOC approval as a medical product in the US	Increase in wound infections when used time consuming therapy (full debridement within 24 h)
